# Pan-cancer network disorders revealed by overall and local signaling entropy

**DOI:** 10.1093/jmcb/mjab031

**Published:** 2021-06-07

**Authors:** Li Feng, Yi-Di Sun, Chen Li, Yi-Xue Li, Luo-Nan Chen, Rong Zeng

**Affiliations:** 1 CAS Key Laboratory of Systems Biology, CAS Center for Excellence in Molecular Cell Science, Institute of Biochemistry and Cell Biology, Chinese Academy of Sciences, Shanghai 200031, China; 2 University of Chinese Academy of Sciences, Shanghai 200031, China; 3 Institute of Neuroscience, CAS Center for Excellence in Brain Science and Intelligence Technology, Chinese Academy of Sciences, Shanghai 200031, China; 4 Center for Single-Cell Omics, School of Public Health, Shanghai Jiao Tong University School of Medicine, Shanghai 200025, China; 5 Bio-Med Big Data Center, CAS Key Laboratory of Computational Biology, CAS–MPG Partner Institute for Computational Biology, Shanghai Institute of Nutrition and Health, University of Chinese Academy of Sciences, Chinese Academy of Sciences, Shanghai 200031, China; 6 CAS Key Laboratory of Systems Biology, Hangzhou Institute for Advanced Study, University of Chinese Academy of Sciences, Chinese Academy of Sciences, Hangzhou 310024, China

**Keywords:** pan-cancer, network, entropy

## Abstract

Tumor development is a process involving loss of the differentiation phenotype and acquisition of stem-like characteristics, which is driven by intracellular rewiring of signaling network. The measurement of network reprogramming and disorder would be challenging due to the complexity and heterogeneity of tumors. Here, we proposed signaling entropy (SR) to assess the degree of tumor network disorder. We calculated SR for 33 tumor types in The Cancer Genome Atlas database based on transcriptomic and proteomic data. The SR of tumors was significantly higher than that of normal samples and was highly correlated with cell stemness, cancer type, tumor grade, and metastasis. We further demonstrated the sensitivity and accuracy of using local SR in prognosis prediction and drug response evaluation. Overall, SR could reveal cancer network disorders related to tumor malignant potency, clinical prognosis, and drug response.

## Introduction

Cancer is considered a result of a series of driving mutations obtained by one or several clones to make the cells adaptive and reproductive (Hahn and Weinberg, 2002; Martincorena et al., 2017). Epidemiological studies have shown that the process of tumor development can be as long as several decades and a solid tumor undergoes up to eight rate-limiting events from the occurrence to clinical detection (Armitage and Doll, 1954). Meanwhile, the tumor is not a unitary environment because of tumor heterogeneity (Hanahan and Weinberg, 2011; Prasetyanti and Medema, 2017; Reiter et al., 2019). The existence of subpopulations and the dynamic relationship and interaction among subpopulations lead to the complex state of this microenvironment. Therefore, sensitive indicators are needed to assess the complexity of tumor microenvironment. Exploring the status of tumor development not only help us understand the causes of tumorigenesis and find the key signaling pathways but also contribute to the personalized and precise treatment, including targeted drugs.

In addition, the development of cancer can be seen as a process in which cells gradually lose differentiation phenotype and obtain stem-like features or ‘stemness’ that is defined as the potential of self-renewal and differentiation from the cell of origin. Cells with the highest level of stemness possess the ability to give rise to all cell types in the adult organism. Tumors with high differentiation potential are more likely to spread to distant organs, causing disease progression and poor prognosis, particularly because metastases are usually resistant to available therapies (Visvader and Lindeman, 2012; Friedmann-Morvinski and Verma, 2014; Ge et al., 2017; Shibue and Weinberg, 2017). Therefore, tumor heterogeneity can also be attributed to the fact that tumor tissues have different degrees of differentiation potential. Malta et al. (2018) proposed an index to identify stemness features associated with oncogenic differentiation using machine learning method. However, this method was trained only in stem cells. Considering that the complexity or disorder of tumor is caused by multiple factors including heterogeneity and stemness, a more robust and informative indicator is needed.

At present, studies characterized for cancer often focus on the change of one or several key genes. The discovery of driver mutations is of great significance and has been applied in drug discovery, such as imatinib that targets BCR–ABL fusion, gefitinib that binds to and inhibits EGFR, and trastuzumab that inhibits HER2 (Carter et al., 2009; Pleasance et al., 2010; Vogelstein et al., 2013). However, considering the existence of tumor heterogeneity, single driver mutation is difficult to explain the overall change. Even the well-known driver mutation on BRAF gene (e.g. V599E) was only present in 66% but not all of malignant melanomas (Davies et al., 2002). Previous studies have also shown that cancer is a system-level network change by accumulation of genetic and/or epigenetic changes under the molecular network structure (Pe'er and Hacohen, 2011; Creixell et al., 2015). Therefore, the global network characteristics are of particular importance. The biological network analysis could help us understand the relationship between biological network and cancer development and thus provide clues for clinical treatment (Ozturk et al., 2018). For example, a network analysis revealed the underlying molecular, tumor type-specific networks accounted for different responses to the inhibitor targeting BRAF V600E in melanoma and colorectal cancer. The inferior response in colorectal cancer is caused by feedback activation of EGFR, which maintains cell proliferation in the presence of the inhibitor (Prahallad et al., 2012). In the field of cancer network analysis, statistical characteristics of the network are also of help to understand the occurrence and development of cancer in depth (Platzer et al., 2007), which are always quantitative features that allow for comparison between samples and contain more information without feature selection. Some network features, such as network entropy or signaling entropy (SR) (Teschendorff and Severini, 2010; West et al., 2012; Teschendorff et al., 2014; Cheng et al., 2016), have also been proposed. Entropy is a measure of the degree of disorder in the system, and network entropy or SR, as a global network characteristic, reflects the uncertainty of signal transmission in the molecular interaction network. In 2012, West et al. (2012) found that cancer samples often have higher network entropy values than normal controls using a network integrating microarray transcriptomic data and protein–protein interaction (PPI) network. Banerji et al. (2015) found that SR can be a prognostic measure by analyzing 3668 breast cancer and 1692 lung adenocarcinoma samples. Cheng et al. (2016) used a similar method to calculate network entropy and found that it can be used to characterize tumor progression and anticancer drug responses. In 2017, SR was used to analyze single-cell data and the results showed that SR can reflect the stemness of the cell (Teschendorff and Enver, 2017). According to previous studies, SR has been recognized as a quantitative measure to assess different cancer states, but the conclusion was made by limited samples or cancer types. Besides, SR showed limited value in prognosis prediction (Teschendorff and Severini, 2010; West et al., 2012; Banerji et al., 2015).

Here, we applied and explored the concept of SR (Teschendorff and Enver, 2017) in more extensive samples and more diverse data types to measure tumor development and classify cancer subtypes. We also investigated the role of local entropy in prognosis prediction and the underlying mechanism of high-entropy cancer. Our study also shows the correlation between SR and drug response, which provides a guidance for drug selection in clinical application.

## Results

### SR as an effective network biomarker

**Figure 1:**
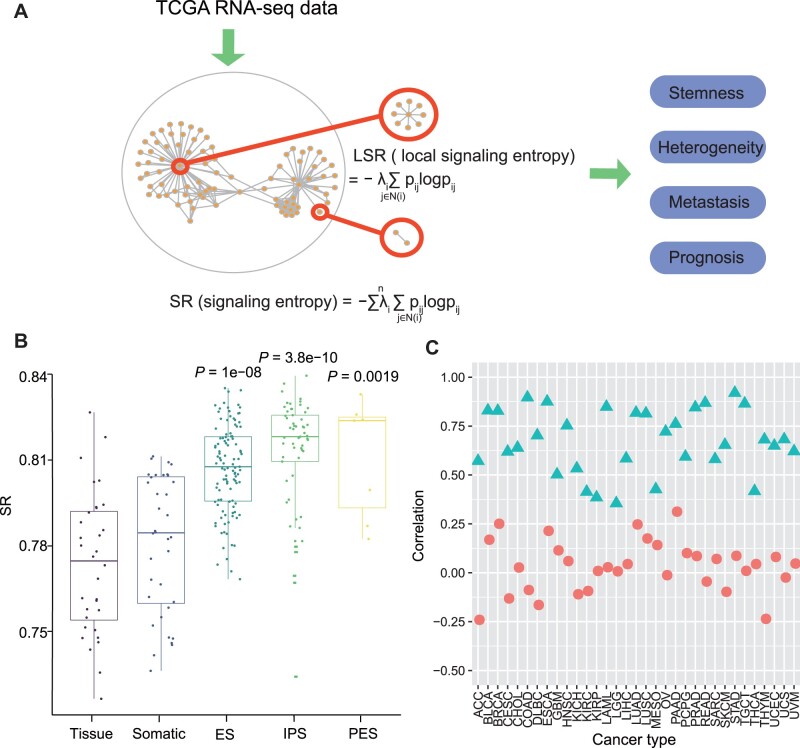
Calculation and validation of SR. (**A**) Overview of SR. The concept of global SR and LSR. SR was explored for the association with stemness index, heterogeneity index, cancer metastasis, and prognosis. (**B**) SR of cells with different differentiation probabilities. The *P*-value (Student’s *t*-test) above each boxplot indicates whether there is a difference between this group and the tissue group. (**C**) The correlation coefficients between SR and mRNAsi (green triangle) or MATH (red circle) in 33 tumor types of TCGA database. ACC, adrenocortical cancer; BLCA, bladder cancer; BRCA, breast cancer; CESC, cervical cancer; CHOL, bile duct cancer; COAD, colon cancer; DLBC, large B-cell lymphoma; ESCA, esophageal cancer; GBM, glioblastoma; HNSC, head and neck cancer; KICH, kidney chromophobe; KIRC, kidney clear cell carcinoma; KIRP, kidney papillary cell carcinoma; LAML, acute myeloid leukemia; LGG, low grade glioma; LIHC, liver cancer; LUAD, lung adenocarcinoma; LUSC, lung squamous cell carcinoma; MESO, mesothelioma; OV, ovarian cancer; PAAD, pancreatic cancer; PCPG, pheochromocytoma & paraganglioma; PRAD, prostate cancer; READ, rectal cancer; SARC, sarcoma; SKCM, melanoma; STAD, stomach cancer; TGCT, testicular cancer; THCA, thymoma; THYM, thymoma; UCEC, endometroid cancer; UCS, uterine carcinosarcoma; UVM, ocular melanomas.

To calculate SR, we firstly constructed a probabilistic signaling network by combining PPI network with gene expression profiles. The nodes of the network represent different genes, and edges indicate interactions between pairs of genes. The weight of each edge represents the possibility of connecting two genes, which are proportional to the expression of the two nodes that are connected (Figure 1A; Materials and methods section). Basically, the probabilistic signaling network assumes that highly expressed genes are more likely to be connected in the signaling network (Banerji et al., 2015; Teschendorff and Enver, 2017). Local signaling entropy (LSR) was calculated for each node by combining all the normalized weights of edges around it (Figure 1A; Materials and methods section). The sum of all LSR values in the network is termed as SR. We compared SR with previously reported diagnostic and prognostic markers including stemness and heterogeneity (Teschendorff and Severini, 2010; Hanahan and Weinberg, 2011; Banerji et al., 2015) and also explored the role of SR in clinical outcome prediction (Figure 1A).

To investigate whether SR could reflect stemness of tissues or cell lines, we calculated SR of samples with different differentiation potentials using data from a study before (Nazor et al., 2012). Not surprisingly, samples with the highest differentiation level demonstrated the lowest SR, whereas embryonic stem cell, induced pluripotent stem cell, and parthenogenetic embryonic stem cell with high differentiation potential showed significantly higher SR (Figure 1B). This result was consistent with a previous study (Teschendorff and Enver, 2017) that SR was a measure of differentiation potential for single cells. We next quantitatively evaluated the relationship between SR and the index ‘mRNAsi’ (Malta et al., 2018), which was reported as an evaluation indicator for tumor stemness. ‘mRNAsi’ was calculated using an innovative one-class logistic regression machine learning algorithm to extract transcriptomic feature sets derived from non-transformed pluripotent stem cells and their differentiated progeny (Malta et al., 2018). SR showed strong correlation with ‘mRNAsi’ in 33 tumor types in The Cancer Genome Atlas (TCGA) database (Figure 1C;Supplementary Table S1). Similarly, we also compared SR with mutant-allele tumor heterogeneity (‘MATH’), a previously reported indicator for tumor heterogeneity (Mroz et al., 2015). ‘MATH’ was calculated from the distribution of mutant allele frequency (MAF) and normalized by the median MAF value to correct for normal DNA in tumor sample (Mroz et al., 2015). An intermediate level of correlation was observed between SR and ‘MATH’ (Figure 1C;Supplementary Table S2). These results indicate that SR can be an effective network biomarker to measure the stemness or differentiation potential of cells.

### SR of different cancer types

**Figure 2:**
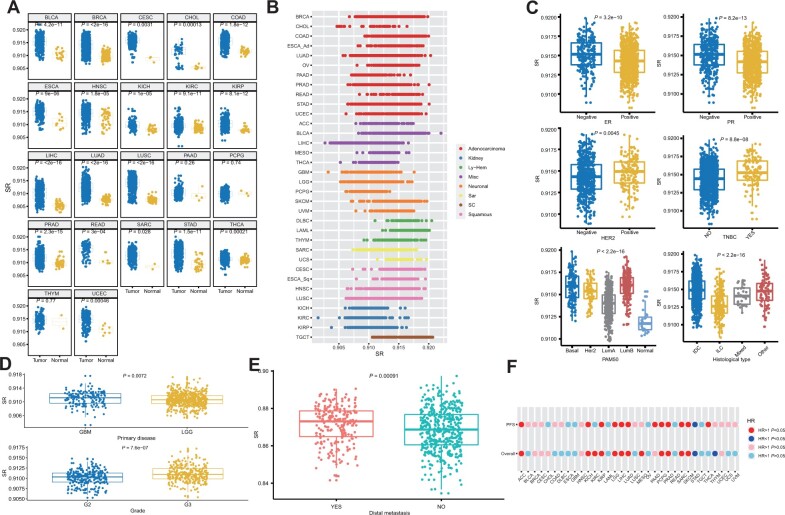
Molecular and clinical features associated with SR. (**A**) SR is higher in tumors in comparison to normal samples in 33 cancer types of TCGA. (**B**) The distribution of SR in 33 TCGA tumor types. The tumor types were grouped based on their histology and cell of origin into SC, Ly–Hem, Adenocarcinoma, Squamous, Neuronal, Sar, Kidney, and Misc. Each spot represents SR of a certain case in the given tumor type. (**C**) Distribution of SR in individual samples with BRCA, stratified by ER, PR, HER2, TNBC, PAM50, and histological type. (**D**) Distribution of SR in patients with GBM and LGG, stratified by primary disease and tumor grade. (**E**) Distribution of SR in samples of breast cancer. SR is higher in samples with distal metastasis in comparison to those without distal metastasis. (**F**) The relationship between SR and prognosis. Hazard ratio (HR) >1 denotes a trend toward higher SR with worse outcome, marked as red (*P *<* *0.05) or pink (*P *>* *0.05) spots. HR < 1 denotes a trend toward higher SR with better outcome, marked as blue (*P *<* *0.05) or skyblue (*P *>* *0.05) spots. Patient outcome is indexed by progression-free survival (PFS, top lane) or overall survival (OS, bottom lane).

The transformation of normal cells into tumor cells is a process gradually losing the differentiation phenotype and obtaining stem cell characteristics (Visvader and Lindeman, 2012; Friedmann-Morvinski and Verma, 2014; Ge et al., 2017). If the determination of lineage is regarded as a probabilistic process, the choice of lineage in normal cells is deterministic, because differentiation leads, by necessity, to activation of specific transcription factors and pathways and thus to a lowering in the uncertainty of signaling patterns, resulting in a lowering of entropy (Teschendorff et al., 2014). Tumor cells are supposed to have higher uncertainty or entropy as they have similar characteristics with stem cells. With the increase of cancer malignancy, the differentiation phenotype is further lost, and the uncertainty of lineage selection is gradually increasing. Considering the role of SR in measuring the differentiation ability of cells, we next applied SR to assess the degree of cancer malignancy. We calculated SR of 10459 patients from 33 cancer types and 678 normal controls in TCGA database based on transcriptomic data. The SR values of cancer samples were significantly higher than that of normal samples (Figure 2A), which was consistent with our assumption. Besides, SR varied a lot in different cancer types (Figure 2B). Compared with other tumor types, germ cell tumors (e.g. TGCT), which is a kind of stem cell-like (SC) tumor, and lympho–hematopoietic (Ly–Hem) tumors (e.g. DLBC, LAML, and THYM) tended to have higher SR values (Figure 2B). When we divided all the samples based on their histology and cell of origin into eight cancer type groups, including SC, Ly–Hem, adenocarcinomas (Adenocarcinoma), squamous cell carcinomas (Squamous), neuronal lineage (Neuronal), sarcomas (Sar), kidney tumors (Kidney), and not belonging to any of the above (Misc), significant differences were observed among groups except for Ly–hem vs. SC, Misc vs. Ly–hem, and Adenocarcinoma vs. Sar (Supplementary Table S7). Hierarchical clustering using SR of different cancer types showed that the three cancer types belonging to the Kidney group (KICH, KIRC, and KIRP) were clustered relatively close, and TGCT was clustered together with a Ly–Hem tumor LAML (Supplementary Figure S5A). In addition, SR distribution patterns of the three renal cell carcinomas (KICH, KIRC, and KIRP) were similar (0.91 ± 0.002, 0.91 ± 0.001, and 0.91 ± 0.002, respectively; Figure 2B), suggesting that SR could distinguish the histological type of cancer and the source of cancer cells to a certain extent.

To further explore the possibility of using SR to predict tumor malignancy, we performed analysis in patients with breast invasive cancer (BRCA) or glioma cancer (GBM and LGG). We found a close relationship between SR and clinical characteristics in BRCA (Figure 2C;Supplementary Figure S2A). By performing k-means clustering using SR of BRCA samples, four clusters were obtained. Chi-square test showed that the four identified clusters were significantly related to various clinical characteristics (Supplementary Figure S5C). Patients with high entropy were more likely to have negative estrogen receptor (ER), negative progesterone receptor (PR), and positive HER2 receptor (Figure 2C). Consistently, the entropy of triple-negative breast cancer (TNBC) was significantly higher than that of non-TNBC (Figure 2C). Besides, the highest SR value was observed in the basal subtype (Figure 2C), the most invasive subtype with the highest degree of malignancy and the worst survival probability among all BRCA subtypes (Perou et al., 2000; Sorlie et al., 2001; Parker et al., 2009). In addition, invasive lobular breast cancer that has a better prognosis showed significantly lower entropy than invasive ductal breast cancer (Figure 2C). Similarly, we found a strong correlation between entropy and pathological grade or histological subtype of glioma (Figure 2D;Supplementary Figure S2B). As a more malignant type of cancer, GBM demonstrated significantly higher SR than LGG, suggesting a role of SR in prognosis prediction (Figure 2D; Ceccarelli et al., 2016).

### SR is associated with cancer prognosis and metastasis

To examine the relationship between SR and clinical prognosis, we next evaluated whether SR could predict overall survival (OS) and progression-free survival (PFS) in each cancer type. Not surprisingly, higher SR demonstrated a risk factor for both OS and PFS in most cancer types including ACC, KIRP, LGG, LIHC, LUAD, PCPG, PRAD, SARC, and SKCM (Figure 2F), indicating that patients with high entropy values are prone to have disease progression and worse survival probability. However, we also observed an opposite phenomenon in STAD where higher SR was correlated with better survival, suggesting that the prognosis prediction by SR is affected by the type of cancer (Figure 2F). In addition to prognosis, a significant correlation between entropy and metastasis was observed in the independent breast cancer data from a previous research (Yau et al., 2010). Patients with distal metastasis demonstrated higher entropy values (Figure 2E), and patients with high SR (median split) were more likely to have metastasis given certain period (Supplementary Figure S2C). Moreover, we obtained similar results in another dataset (Yu et al., 2004; Chandran et al., 2007) with 683 breast cancer samples (Supplementary Figure S2E). To validate this association, we next retrieved a single-cell sequencing dataset of breast cancer (GSE75688) (Chung et al., 2017) and observed that single cells from metastatic sites had higher entropy values than those from primary tumors (Supplementary Figure S2D). However, entropy values of individual cells varied even in the same tissue from the same patient, indicating the presence of intra-tumor heterogeneity. These results suggest that SR is a measure for the degree of cancer malignancy, demonstrating its role in the prediction of clinical prognosis and metastasis.

### SR based on proteomic data

**Figure 3:**
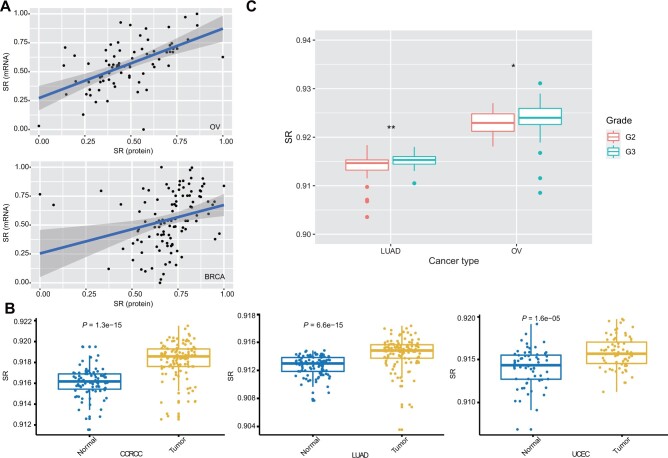
SR based on proteome. (**A**) Correlation between SR based on transcriptomic and proteomic data in BRCA and OV. (**B**) SR based on proteome is higher in tumors in comparison to normal samples in clear cell renal cell carcinoma (CCRCC), LUAD, and UCEC. (**C**) Association between tumor grade and SR based on proteomic data in LUAD and OV. SR is higher in samples at grade 3 in comparison to those at grade 2.

Compared to transcriptomic data, proteomic data reflect pathway activity of an organism or the response to external treatment more accurately, as protein is the performer of biological function. We evaluated SR of various cancer types using multiple proteomic datasets in CPTAC database (https://proteomics.cancer.gov/programs/cptac) and found a high consistency between SR based on proteomic and transcriptomic data in BRCA (*r* = 0.27, *P *=* *0.004) and OV (*r* = 0.46, *P *=* *2.945e−05; Figure 3A), indicating that SR is a robust value to reflect the real degree of network confusion with no limit to data type. We next explored the relationship between SR based on proteomic data and clinical characteristics. For three cancer types with normal controls available, a comparison of SR values of tumor and normal samples showed that SR of normal samples was far less than that of tumor samples (Figure 3B), consistent with the results based on transcriptomic data (Figure 2A). In addition, we found that proteome-based SR for grade 3 was significantly higher than that for grade 2 in LUAD and OV (Figure 3C), consistent with the result from transcriptomic data (Figure 2D). Cancer grade is determined by the differentiation degree of tumor cells (Liu, 2018). Therefore, higher differentiation degree represents higher grade in classification, which results in higher SR, just in line with our hypothesis that SR is a standard for measuring the disorder and differentiation degree of tumor cells. To sum up, SR based on proteomic data and that based on transcriptomic data come to the same conclusion, indicating the robustness of this method.

### Distribution of LSR and enrichment analysis

**Figure 4:**
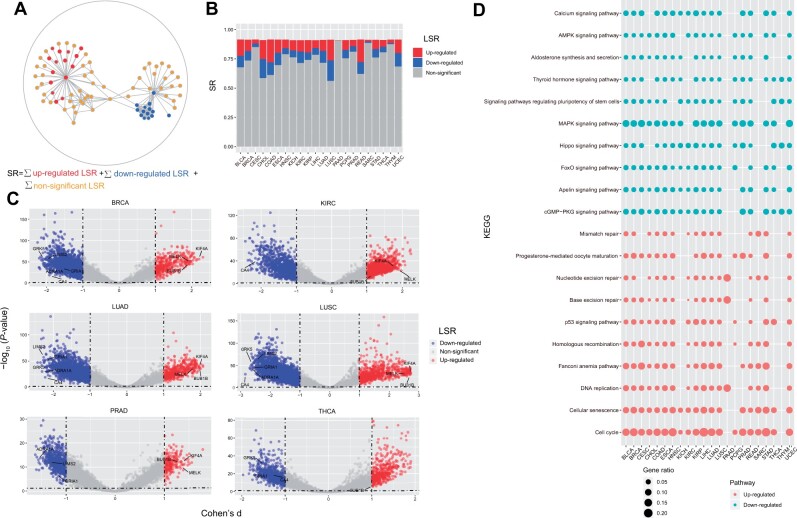
Distribution of LSR and enrichment analysis. (**A**) Concept map of LSR. SR is the sum of LSR, and LSR can be divided into three types according to the differences between tumor and normal samples: upregulated, downregulated, and no significant change in tumor. (**B**) The composition of three kinds of LSR in 22 different cancer types. (**C**) Volcano plots of differential LSR between tumor and normal samples in six cancer types. Effect size Cohen’s d was calculated, and *P*-values were calculated by Student’s *t*-test and adjusted by FDR method. (**D**) KEGG enrichment analysis using differential LSR between tumor and normal samples. The red dots represent pathways derived from upregulated LSR, while the green dots show the pathways with downregulated LSR.

SR represents the overall characteristic of a sample, i.e. the degree of chaos. As SR is obtained by summing up all local entropy values, we next explored the contribution of local entropy to the overall difference. By comparing with normal samples, local entropy can be split into three parts, namely upregulated LSR in the tumor, downregulated LSR, and LSR without significant changes (Figure 4A). There are 22 types of cancer in TCGA database with normal samples as control, and their compositions of the three kinds of LSR are different (Figure 4B). The sum of upregulated LSR varies between 0.002 and 0.195 (ratio ranges from 0.3% to 21.3%), and the sum of downregulated LSR varies from 0.0005 to 0.176 (0.06%–19.2%) (Supplementary Table S3). The extreme values may be caused by a small number of normal samples in some cancer types. For the six cancer types (BRCA, KIRC, LUAD, LUSC, PRAD, and THCA) containing >50 normal samples, BUB1B, KIF4A, and MELK were among the 30 with the most significantly upregulated local entropy of four cancer types (Figure 4C). BUB1B encodes a kinase involved in spindle checkpoints that localizes to the centromere and facilitates the proper separation of chromosomes. According to previous reports, mutations in BUB1B were indeed found in patients with colorectal cancer, but further studies are needed to determine whether the mutations increase its carcinogenicity (Hahn et al., 2016). KIF4A-encoded kinase is involved in intracellular trafficking of membrane organelles and may be involved in the process of mitosis. One study reported that, in liver cancer, upregulation of KIF4A promotes cell proliferation by activating AKT signaling pathway, which makes liver cancer patients have a worse prognosis (Huang et al., 2018). MELK is a potential therapeutic target for cervical cancer and its high expression is associated with poor prognosis in adrenocortical carcinoma (Kiseljak-Vassiliades et al., 2018; Wang et al., 2018). ADRA1A, GRIA1, CA4, GRK5, and LIMS2 were the most common ones with downregulated LSR (Figure 4C). Enrichment analysis using differential LSR showed a strong consistency of pathways enriched in different cancer types. Pathways with the upregulated LSR were mainly in homologous recombination, cell cycle, and DNA repair, consistent with the concept that SR estimates the malignancy of tumor by estimating the differentiation degree. The downregulated LSR-enriched pathways were mainly related to signaling transduction (Figure 4D), suggesting that there may be local barriers in these pathways in cancer, and a decrease in the partial LSR of the pathway means a decrease in connectivity and interaction with surrounding genes, leading to abnormal access to the pathway. From the microscopic perspective, LSR deconstructs the overall difference. High SR is usually caused by the upregulation of signaling pathways related to cell proliferation, while low SR is caused by the abnormality of transduction-related pathways.

Both SR and LSR are related to clinical characteristics. SR is an overall value, which reflects the degree of disorder of the samples. High SR is associated with high malignant degree or high pathological grade of tumor samples, which are prone to metastasis and poor prognosis. LSR could further quantify and explain such correlation. Hierarchical clustering using LSR showed that the three cancer types belonging to the Kidney group (KICH, KIRC, and KIRP) were clustered together, TGCT was clustered together with the other two types of Ly–Hem tumors, and most Adenocarcinomas tumors were clustered together (Supplementary Figure S5B). Four clusters obtained by clustering using LSR matrix were significantly correlated with a variety of clinical features, including ER-positive, PR-positive, HER2-positive, and PAM50 subtypes, and the second cluster was significantly lower than the other three (*P *<* *2.2e−16) (Supplementary Figure S5D).

### Clinical prognosis prediction by LSR

**Figure 5:**
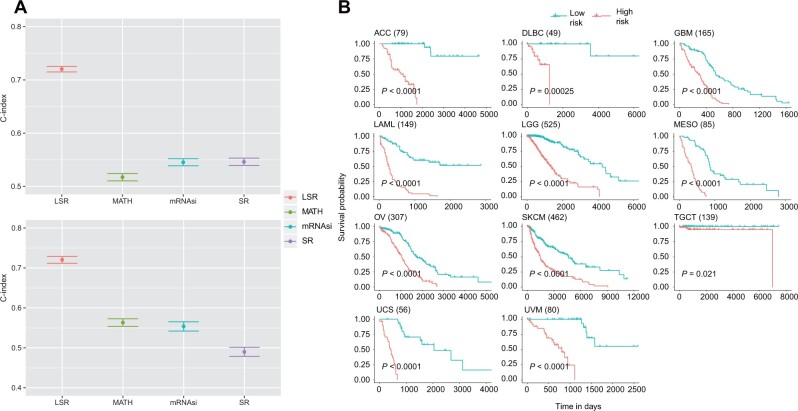
LSR is used to predict patients’ survival. (**A**) Top: C-indexes of LSR, MATH, mRNAsi, and SR in 22 types of cancer with normal samples available. Bottom: C-indexes of LSR, MATH, mRNAsi, and SR in the other 11 types of cancer without normal samples. (**B**) Survival curves of two groups stratified by LSR. High-risk group (red) was predicted to have poor survival, and low-risk group (green) to the opposite. The total number of the patients with the particular tumor for survival analysis is in parentheses.

SR is associated with OS and PFS (Figure 2F), and in most cancer types, higher SR is associated with poorer OS and PFS probability. However, not all cancer types have such a pattern, indicating the complexity and heterogeneity of cancer. Thus, we next explored applying LSR in clinical prognosis prediction. We constructed proportional hazard regression models using LSR as the feature, and the consistency index (C-index) was used to evaluate the predictive ability of each model. For the choice of LSR features, we only included those differentially expressed in cancer compared with normal samples. Briefly, we firstly calculated the differential LSR values in 22 kinds of cancer separately, and then took the common LSR features in 16 kinds of cancer, including 38 upregulated and 14 downregulated. We next built predictive models with these 52 features for the survival of patients from 22 cancer types, and the predicted result was significantly better than the model constructed only with SR, mRNAsi, or MATH (Figure 5A). Then, we applied the model built with 52 LSR features to predict the survival of the other 11 cancer types, which also performed better than other models (Figure 5A). We calculated the Cox proportional hazard ratio for each patient and divided patients into high-risk and low-risk groups based on median split. For the 11 cancer types without control, the survival of the high-risk group was indeed significantly worse than that of the low-risk group (Figure 5B). Since the number of samples was smaller than that of features, we excluded two tumor types. Of the remaining 31 tumor types, the survival of the high-risk group we predicted was still significantly worse than that of the low-risk group (Supplementary Table S4). Although SR has been associated with a variety of cancer characteristics and, to some extent, can be an indicator of patients’ prognosis, using LSR would help us make better prognosis prediction and gain insight into the underlying causes for different patients’ outcomes.

### Drug response prediction with LSR

Although there are many indicators to measure the malignant degree of cancer, few single clinical indicators or gene expression features can predict the responses of cancer patients to drugs. Although gene mutations guided drug usage to certain extent, unfortunately, the complexity of cancer determines its strong limitations. Therefore, we next explored the role of SR, especially LSR, in the guidance of drug use for cancer patients.

**Figure 6:**
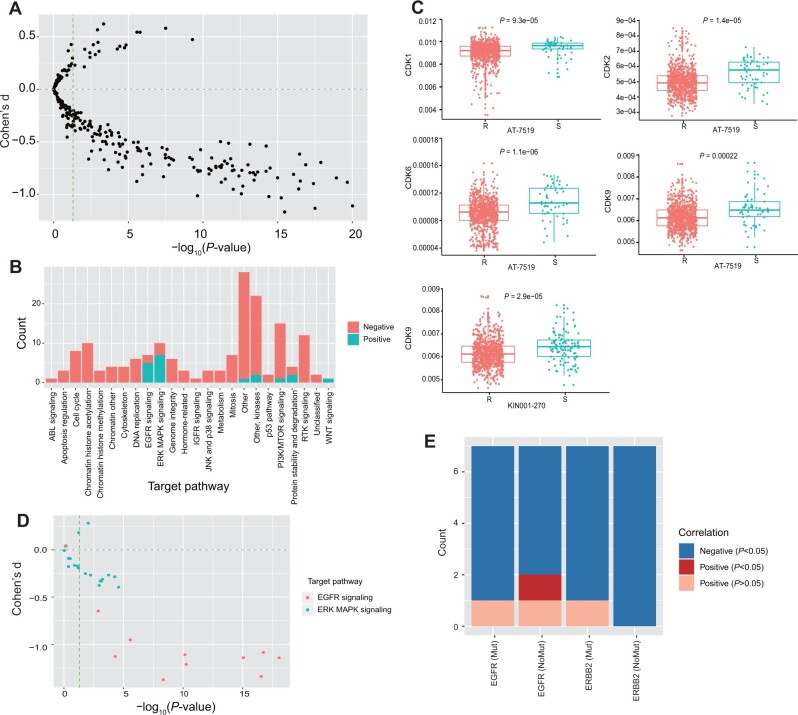
SR for prediction of drug response. (**A**) Differences of SR between sensitive and resistance cell lines for 265 drugs. Each point indicates a drug; positive effect size means that SR is higher in resistance cell line. *P*-values were calculated by Student’s *t*-tests. (**B**) Pathways targeted by drugs with significant differences between sensitive and resistance cell lines. Effect size Cohen’s d is positive (red) or opposite (green). (**C**) LSR values are higher in sensitive cell lines to drugs AT-7519 and KIN001-270. (**D**) Differences in LSR ratio between sensitive and resistance cell lines to drugs targeting EGFR and ERK MAPK signaling pathways. Each point indicates a drug; positive effect size Cohen’s d means that SR is higher in resistance cell line. *P*-values were calculated by Student’s *t*-tests. (**E**) Correlation between LSR ratio and IC50. Red means significant positive correlation (correlation coefficient > 0, *P *<* *0.05), blue means significant negative correlation (correlation coefficient < 0, *P *<* *0.05), and light red means non-significant positive correlation (correlation coefficient > 0, *P *>* *0.05). Count is the number of corresponding drugs. *P*-values were calculated by Pearson correlation test.

In order to study the relationship between entropy and drug response, we retrieved gene expression and drug response data of 265 drugs and 1018 cell lines from GDSC database (https://www.cancerrxgene.org/). We calculated the SR based on transcriptomic data for each cell line. For each drug, we divided cell lines into resistant and sensitive groups according to IC50 value, using the criteria from previous study (Iorio et al., 2016; see Materials and methods section), and compared the SR between two groups. The results showed that there was significant difference in SR between resistant and sensitive groups of cell lines for 165 out of 265 drugs, among which the SR of sensitive group was higher than that of resistant group for 146 drugs and opposite for the other 19 drugs (Figure 6A). For drugs demonstrating significant difference in SR between resistant and sensitive cell lines, we next focused on the associated pathways of the drug-targeted genes. Except for drugs targeting EGFR and ERK MAPK signaling pathways, where the SR was higher in resistant cell lines, the SR in sensitive cell lines was basically higher for other target pathways (Figure 6B). Taking the cell cycle pathway as an example, the higher the SR, the more sensitive the cells to related drugs (Figure 6B). This could possibly be explained by that the greater degree of cell differentiation in cell lines with higher SR provides a suitable environment for cell cycle inhibitors to respond to drugs. For local entropy, the LSR values of genes targeted by drugs were also higher in sensitive cell lines than in resistant cell lines. AT-7519 is an inhibitor targeting multiple cyclin-dependent kinases including cyclin-dependent kinase 1 (CDK1), CDK2, CDK6, and CDK9. The LSR values of these four kinases were significantly higher in sensitive cell lines (Figure 6C). Similarly, KIN001-270 is a CDK9 inhibitor, and the LSR values of CDK9 were also higher in its sensitive cell lines (Figure 6C). As drugs targeting EGFR and ERK MAPK signaling pathways have limited targets, we calculated the LSR ratio by dividing the LSR of each target by the total SR to eliminate the influence of other genes. The results showed that the LSR ratios in almost all the sensitive cell lines were higher than in the resistant cell lines (Figure 6D).

To further demonstrate the role of SR in drug response prediction, we compared the SR with existing signatures like gene mutations, which often play an important role in drug response. Taken drugs targeting EGFR signaling pathway as an example, EGFR or ERBB2 mutation could not accurately distinguish whether a cell line is resistant or sensitive to the corresponding drugs, while cell lines with smaller IC50 tend to have larger LSR ratio of EGFR (Supplementary Figure S4 and Table S5). We observed more negative correlations between LSR ratio and drug response than positive correlations, no matter whether EGFR or ERBB2 was mutated or not. In other words, the higher the LSR ratio is, the lower the IC50 value is and more sensitive the cell line is to the corresponding drug. In particular, in the cell lines without mutations, we still observed a significant negative correlation between LSR ratio and IC50 value (Figure 6E). Therefore, LSR ratio proposed by us has a great potential to predict drug response and provide medication guidance to patients without relevant mutations.

## Discussion

We applied SR to assess the degree of disorder of the tumor, which is the result of a combination of various factors such as stemness and heterogeneity of the tumor. The level of SR is related to various clinical features of the tumor and can be used in medication as a supplement of genomic variation. Local entropy derived from SR can be used to predict the survival of tumor patients. Overall, SR can help us understand the occurrence and development of tumors and achieve more accurate treatment for patients.

SR has significant correlations with stemness in various tumor types (Figure 1C;Supplementary Figure S1). In 33 tumor types, the average value of the correlation coefficient between SR and mRNAsi reaches 0.67. The correlation coefficient is as high as 0.92 in STAD and exceeds 0.8 in 11 tumor types. On the other hand, the correlation coefficient between SR and MATH varies a lot and is positively correlated only in 22 tumor types (Figure 1C;Supplementary Figure S2). The correlation between SR and mRNAsi/MATH is independent and there is no synergy. SR is a quantitative indicator that can reflect the degree of disorder of the tumor. Compared with mRNAsi, it does not require feature selection and also buries more information. The large difference between SR and MATH may be caused by the distinct data types used for their calculation; MATH is obtained through the somatic mutation of genome, and SR is obtained using transcriptomic data. Some previous studies (Peiris-Pages et al., 2016; Li et al., 2017; Zheng et al., 2018) have used buck or single-cell RNA sequencing data to explore the heterogeneity of tumors and tumor stem cells, but most of them are qualitative instead of quantitative indicators. SR as a quantitative indicator can reflect tumor heterogeneity and stemness and helps us better grasp the disease development of cancer patients and understand the mechanism of tumor development.

SR, as a measure of the overall state, includes complex information and is not very suitable for prognosis prediction. Therefore, we select LSR for predicting the survival of cancer patients, and we found it performing well in predicting the survival for various tumor types no matter whether control exists, which is far better than the other three indicators. Furthermore, accurate prognostic prediction is achieved without machine learning. Another advantage of using local entropy for prediction is that the intersection of local entropy in different tumor types is relatively large, demonstrating the wide applicability of SR in prognosis prediction. By contrast, quite a few tumor prognostic markers are often only suitable for certain types of cancer, such as breast cancer (Braden et al., 2014), lung cancer (Woodard et al., 2016), glioblastoma (Salvucci et al., 2019), and hepatocellular carcinoma (Teufel et al., 2019), and are not universal markers of pan-cancer. There are also some studies that use large samples of pan-cancer data to find more applicable tumor prognostic markers. Gentles et al. (2015) present a pan-cancer resource and meta-analysis of expression signatures from ∼18000 human tumors with OS outcomes across 39 malignancies. Through clustering, they discovered that one cluster is broadly associated with inferior outcomes and is functionally linked to cell proliferation and cell cycle phase, which is consistent with our results. Compared to normal tissues, genes with higher local entropy in tumor tissues are enriched in the cell cycle pathway; among the 52 genes we used to predict survival, 8 genes (BUB1B, BUB1, DBF4, E2F3, CDC45, MCM3, PLK1, PKMYT1) are related to the cell cycle pathway. There are also some differences between the study and our research. They used univariate Cox regression to find genes related to survival and directly used the transcriptomic data (Gentles et al., 2015), while we used the LSR features. LSR itself includes the relationship among genes, which can be regarded as the overall feature of small networks with a single gene at the core, and therefore it contains more information, which helps to discover the core gene related to survival that cannot be found when using the expression value of a single gene alone. Moreover, we conducted multivariate Cox regression. Some of the features are not significantly related to survival in one tumor type but are significantly related to survival in another tumor type. We retain these features to ensure that the common characteristics related to survival can be kept to the greatest extent and realize the prediction of pan-cancer survival.

SR reflects the disorder degree of whole network and is the sum of LSR. However, for the efficacy of targeted therapy, local network alteration is often more important than the change of whole network. Therefore, LSR is more accurate than SR in some aspects, such as predicting the sensitivity of cell lines to drugs (Figure 6C‒E). In this study, we focused on drugs that target the cell cycle and EGFR pathways. Drugs that target the cell cycle pathway, such as AT-7519 and KIN001-270, specifically target CDKs. These kinases and their regulated cyclin partners play a central role in the growth, division, and death of eukaryotic cells. Although we hope that SR can be used as a universal measure of drug efficacy, we must admit that, according to the mechanism of drug action, SR has a different effect on predicting drug responses. This is caused by SR itself, which is determined by the characteristics of the cell. According to the differential local entropy analysis between normal and tumor samples, we can see that the difference in SR between the two groups is caused in part by genes belonging to pathways such as cell cycle, DNA replication, and homologous recombination (Figure 4D). Higher SR means the local networks centered of these genes (including CDKs) are overactive, providing a suitable target aim and environment for the drug to function. Therefore, it is more accurate to use SR to predict the response of this kind of drugs, and the higher SR in sensitive cell lines using these drugs is indeed seen in the GDSC database (Figure 6B). For drugs targeting the EGFR pathway, SR is not sufficient to predict the efficacy of drugs. The specific proportion is not large, and thus it is necessary to focus on local entropy as a complement. EGFR belongs to the receptor tyrosine kinase of the HER/ERBB family, which includes HER1 (EGFR/ERBB1), HER2 (ERBB2), HER3 (ERBB3), and HER4 (ERBB4) (Wells, 1999). EGFR tyrosine kinase activity may be regulated by multiple carcinogenic mechanisms such as EGFR gene mutation, increased gene copy number, and overexpression of EGFR protein (Ciardiello and Tortora, 2008). Receptors and ligands also mediate complex interactions between tumor cells and the tumor microenvironment. Improper activation of EGFR tyrosine kinase activity inhibits tumor cell apoptosis and promotes tumor progression (Woodburn, 1999). EGFR may also interact with the integrin pathway (Hazan and Norton, 1998; Herbst and Bunn, 2003), activate matrix metalloproteinases, change cell adhesion, stimulate cell viability and invasion, and promote metastasis (Ellerbroek et al., 2001). These findings make EGFR a reasonable therapeutic target and support the development of new anti-cancer drugs against EGFR. Many existing studies focus on the effect of EGFR mutations on the efficacy of EGFR tyrosine kinase inhibitors (EGFR-TKIs) when judging whether to use EGFR inhibitors in the clinical treatment (Gazdar, 2009; Ju et al., 2016; Ou et al., 2017). However, according to Paez et al. (2004), only 21% of patients with lung adenocarcinomas have EGFR mutations. Are EGFR-TKIs still or not applicable to patients without relevant mutations? Our analysis shows that regardless of whether the patient has a mutation or not, IC50 is negatively correlated with the LSR ratio of EGFR, which means that patients without EGFR mutation but with high LSR ratio of EGFR may also be suitable for EGFR-TKI treatment. Although we can only draw preliminary conclusions based on cell line results due to lack of patient medication data, this undoubtedly provides a treatment possibility for patients who have not detectable EGFR mutations.

SR using transcriptomic and proteomic data is a biomarker to measure the development of the tumor and can also be applied in clinical treatment with good robustness and wide applicability. Compared to overall SR, LSR shows more accuracy in predicting patient survival and drug responses, which provides clues for clinical treatment.

## Materials and methods

### Data collection and pre-processing

Transcriptomic data include the RNA sequencing data from TCGA database and microarray data from GEO database. TCGA data were download from https://xenabrowser.net/datapages, including 33 cancer types. RSEM value was used to quantify the gene expression of RNA sequencing data and applied to calculate SR. All microarray data analyzed in this work are publicly available from the following GEO (www.ncbi.nlm.nih.gov/geo/) accession numbers: GSE2034, GSE5327, GSE6605, GSE6606, GSE7390, and GSE75688. All proteomic data were downloaded from the CPTAC database (https://proteomics.cancer.gov/programs/cptac), including five cancer types BRCA (Cancer Genome Atlas Network, 2012), LUAD (Mertins et al., 2018), CCRCC (Clark et al., 2019), OV (Cancer Genome Atlas Research Network, 2011), and UCEC (Dou et al., 2020), where the data were generated by TMT method. The log ratio values in each dataset were normalized by columns and filled by the median of each protein of samples before they were applied to calculate SR.

For drug data, the IC50s were recorded as the natural logarithm of the half-maximal inhibitory μM concentrations. Although the continuous IC50 values were used in the analysis, it is necessary to define a binarization threshold. This threshold is used to divide the cell lines into two classes, the sensitive cell lines and the resistant cell lines, according to the previous study (Iorio et al., 2016), which employed the procedure described in Knijnenburg et al. (2016) to find the binarization threshold for each of the drugs.

### Calculation of SR and LSR

The SR is calculated by firstly making an integrated network, which in this case is the PPI network. The PPI network covering 303600 unique interactions connecting 8434 genes was downloaded from the Human Protein Reference Database (http://hprd.org), which is manually curated by the literature.

Assuming that if both genes are highly expressed, the possibility of their interaction in the network is greater, which is constructed by appealing to a simple version of the mass action principle, namely that the rate of a reaction is proportional to the product of the active masses of the reagents involved. SR is the entropy rate of the probabilistic signal process on the network, which quantifies the speed of the signal propagates throughout the network, thus measuring the number of biological processes that are ‘active’ in some sense. Therefore, normal or less vicious cells activate only specifically in functionally related pathways and are expected to exhibit lower entropy rates because the signals cannot spread to ‘inactive’ regions of the network.

The normalized transcriptomic or proteomic data matrix was used to evaluate the weight of each edge in the PPI network. The weight of gene i and gene j is recorded as w_ij_, and w_ij_ is proportional to the expression of gene i and gene j, i.e. w_ij_ ∼ x_i_x_j_, where w_ij_ represents the possibility of connecting two genes. In other words, if the expression of two genes is high, we think that the possibility of connecting two genes is relatively large. Then, we normalize the weights so that the sum of the weights of each gene was 1, and thus obtain the matrix P:

$$p_{ij}=\frac{x_{j}}{\sum_{k\in N(i)}x_{k}}=\frac{x_{j}}{{\left(Ax\right)}_{i}}$$

where N(i) represents all adjacent genes of gene i and A is the adjacency matrix of the PPI network (A_ij_ = 1 if i and j are connected, 0 otherwise, and with A_ij_ = 0).

The SR:

$$Sr\left(\vec{x}\right)=-\sum_{i=1}^{n}\lambda_{i}\sum_{j\in N(i)}p_{ij}\;\text{log}\;p_{ij}$$

where λ is the invariant measure, satisfying λP=λ and the normalization constraint λ^T^=1. The invariant measure, also known as steady-state probability, represents the relative probability of finding the random walker at a given node in the network (under steady-state conditions, i.e. long after the walk is initiated). Nodes with high values thus represent nodes that are particularly influential in distributing signaling flux in the network. In the steady-state, we can assume detailed balance (conservation of signaling flux, i.e. λ_i_p_ij_=λ_j_p_ji_), and it can be shown (Teschendorff et al., 2014) that λ_i_=x_i_ (Ax)_i_/(x^T^Ax).

We denote this maximum entropy rate by maxSr, which is the logarithm of the maximum eigenvalue of the adjacency matrix, and define the normalized entropy rate (with range of values between 0 and 1) as:

$$SR\left(\vec{x}\right)=\frac{Sr(\vec{x})}{\mathrm{maxSr}}.$$

Since the SR is formed by adding LSR, we define it as:

$$\mathrm{LSR}\left(\vec{x}\right)={\;-\lambda }_{i}\sum_{j\in N(i)}p_{ij}\;\text{log}\;p_{ij}.$$

The calculation method of SR comes from Teschendorff and Enver (2017). In that paper, there is also a calculation method of LSR, which has been modified in this study to be more consistent with the notion that SR is a sum of LSR.

### Hierarchical clustering and k-means clustering

The mean value of SR of the 34 cancer types (originally 33 cancer types; due to the existence of Squamous and Adenocarcinoma subtypes in ESCA, ESCA was divided into two categories and thus a total of 34 cancer types) was calculated and used for hierarchical clustering. The mean value of LSR of each of the 34 cancer types was calculated and used for hierarchical clustering. The distance between two observed values was Euclidean distance, and the clustering method was average linkage method. The k-mean method was adopted to divide the clusters by the SR and LSR matrix of breast cancer, respectively, and the nstart option in the kmeans function was used to help select the appropriate initial configuration. The above clustering was realized by hclust and kmeans functions of R language, respectively.

### Differential LSR enrichment analysis

LSR was calculated for each gene in each sample. Given the huge difference between tumor and normal samples, we explored which LSR features affect the final overall SR. Therefore, we calculated the differential LSR values between tumor and normal samples in 22 tumor types and defined them as differential local signaling entropy (DLSR). Two-sided Students’ *t*-test and FDR multiple adjustment test were used to calculate the DLSR values. At the same time, we calculated the Cohen’s d effect size, choosing LSR with FDR value <0.05 and the absolute value of Cohen's d > 1 as different one. According to whether the value of Cohen’s d is >0, we divided the local entropy of difference into two types, upregulated and downregulated. The R package clusterProfiler was used for enrichment analysis.

The 10 most frequent signaling pathways in 22 tumor types are shown in Figure 4D, and all signaling pathways enriched are listed in Supplementary Table S6.

### Survival analysis

Cox proportional hazard model was used to estimate the hazard ratios and C-index for OS and PFS in different cancer types. Fifty-two different LSR features (38 upregulated and 14 downregulated) existed in at least 16 cancer types were taken as independent variables, and their relationship with survival was explored by Cox proportional hazard model. The samples were divided into two groups with high risk and low risk according to whether the logarithmic hazard ratio of each sample was greater than the median of the logarithmic hazard ratio of all samples.

### Statistical analysis

All the correlation coefficients in this study are Pearson correlation coefficients. Two-sided Students’ *t*-test was used to compare the difference of SR between tumor and normal samples. One-tailed Students’ *t*-test was used to compare the difference of proteomic SR in different pathological grades of the same tumor type.

## Supplementary material

Supplementary material is available at *Journal of Molecular Cell Biology* online. 

**Conflict of interest:** none declared.

## Supplementary Material

mjab031_Supplementary_Data

## References

[mjab031-B1] Armitage P. , Doll R. (1954). The age distribution of cancer and a multi-theory of carcinogenesis. Br. J. Cancer 8, 1–12. 13172380 10.1038/bjc.1954.1 PMC2007940

[mjab031-B2] Banerji C.R. , Severini S., Caldas C., et al (2015). Intra-tumour signalling entropy determines clinical outcome in breast and lung cancer. PLoS Comput. Biol. 11, e1004115. 25793737 10.1371/journal.pcbi.1004115 PMC4368751

[mjab031-B3] Braden A.M. , Stankowski R.V., Engel J.M., et al (2014). Breast cancer biomarkers: risk assessment, diagnosis, prognosis, prediction of treatment efficacy and toxicity, and recurrence. Curr. Pharm. Des. 20, 4879–4898. 24283956 10.2174/1381612819666131125145517

[mjab031-B4] Cancer Genome Atlas Network. (2012). Comprehensive molecular portraits of human breast tumours. Nature 490, 61–70. 23000897 10.1038/nature11412 PMC3465532

[mjab031-B5] Cancer Genome Atlas Research Network. (2011). Integrated genomic analyses of ovarian carcinoma. Nature 474, 609–615. 21720365 10.1038/nature10166 PMC3163504

[mjab031-B6] Carter H. , Chen S., Isik L., et al (2009). Cancer-specific high-throughput annotation of somatic mutations: computational prediction of driver missense mutations. Cancer Res. 69, 6660–6667. 19654296 10.1158/0008-5472.CAN-09-1133 PMC2763410

[mjab031-B7] Ceccarelli M. , Barthel F.P., Malta T.M., et al (2016). Molecular profiling reveals biologically discrete subsets and pathways of progression in diffuse glioma. Cell 164, 550–563. 26824661 10.1016/j.cell.2015.12.028 PMC4754110

[mjab031-B8] Chandran U.R. , Ma C., Dhir R., et al (2007). Gene expression profiles of prostate cancer reveal involvement of multiple molecular pathways in the metastatic process. BMC Cancer 7, 64. 17430594 10.1186/1471-2407-7-64 PMC1865555

[mjab031-B9] Cheng F. , Liu C., Shen B., et al (2016). Investigating cellular network heterogeneity and modularity in cancer: a network entropy and unbalanced motif approach. BMC Syst. Biol. *10(Suppl 3)*, 65. 10.1186/s12918-016-0309-9 PMC5009528 27585651

[mjab031-B10] Chung W. , Eum H.H., Lee H.O., et al (2017). Single-cell RNA-seq enables comprehensive tumour and immune cell profiling in primary breast cancer. Nat. Commun. 8, 15081. 28474673 10.1038/ncomms15081 PMC5424158

[mjab031-B11] Ciardiello F. , Tortora G. (2008). EGFR antagonists in cancer treatment. N. Engl. J. Med. 358, 1160–1174. 18337605 10.1056/NEJMra0707704

[mjab031-B12] Clark D.J. , Dhanasekaran S.M., Petralia F., et al (2019). Integrated proteogenomic characterization of clear cell renal cell carcinoma. Cell 179, 964–983.e31. 31675502 10.1016/j.cell.2019.10.007 PMC7331093

[mjab031-B13] Creixell P. , Reimand J., Haider S., et al (2015). Pathway and network analysis of cancer genomes. Nat. Methods 12, 615–621. 26125594 10.1038/nmeth.3440 PMC4717906

[mjab031-B14] Davies H. , Bignell G.R., Cox C., et al (2002). Mutations of the BRAF gene in human cancer. Nature 417, 949–954. 12068308 10.1038/nature00766

[mjab031-B15] Dou Y. , Kawaler E.A., Cui Zhou D., et al (2020). Proteogenomic characterization of endometrial carcinoma. Cell 180, 729–748.e26. 32059776 10.1016/j.cell.2020.01.026 PMC7233456

[mjab031-B16] Ellerbroek S.M. , Halbleib J.M., Benavidez M., et al (2001). Phosphatidylinositol 3-kinase activity in epidermal growth factor-stimulated matrix metalloproteinase-9 production and cell surface association. Cancer Res. 61, 1855–1861. 11280738

[mjab031-B17] Friedmann-Morvinski D. , Verma I.M. (2014). Dedifferentiation and reprogramming: origins of cancer stem cells. EMBO Rep. 15, 244–253. 24531722 10.1002/embr.201338254 PMC3989690

[mjab031-B18] Gazdar A.F. (2009). Activating and resistance mutations of EGFR in non-small-cell lung cancer: role in clinical response to EGFR tyrosine kinase inhibitors. Oncogene 28(Suppl 1), S24–S31. 19680293 10.1038/onc.2009.198 PMC2849651

[mjab031-B19] Ge Y. , Gomez N.C., Adam R.C., et al (2017). Stem cell lineage infidelity drives wound repair and cancer. Cell 169, 636–650.e14. 28434617 10.1016/j.cell.2017.03.042 PMC5510746

[mjab031-B20] Gentles A.J. , Newman A.M., Liu C.L., et al (2015). The prognostic landscape of genes and infiltrating immune cells across human cancers. Nat. Med. 21, 938–945. 26193342 10.1038/nm.3909 PMC4852857

[mjab031-B21] Hahn M.M. , Vreede L., Bemelmans S.A., et al (2016). Prevalence of germline mutations in the spindle assembly checkpoint gene BUB1B in individuals with early-onset colorectal cancer. Genes Chromosomes Cancer 55, 855–863. 27239782 10.1002/gcc.22385

[mjab031-B22] Hahn W.C. , Weinberg R.A. (2002). Rules for making human tumor cells. N. Engl. J. Med. 347, 1593–1603. 12432047 10.1056/NEJMra021902

[mjab031-B23] Hanahan D. , Weinberg R.A. (2011). Hallmarks of cancer: the next generation. Cell 144, 646–674. 21376230 10.1016/j.cell.2011.02.013

[mjab031-B24] Hazan R.B. , Norton L. (1998). The epidermal growth factor receptor modulates the interaction of E-cadherin with the actin cytoskeleton. J. Biol. Chem. 273, 9078–9084. 9535896 10.1074/jbc.273.15.9078

[mjab031-B25] Herbst R.S. , Bunn P.A. Jr. (2003). Targeting the epidermal growth factor receptor in non-small cell lung cancer. Clin. Cancer Res. 9, 5813–5824. 14676101

[mjab031-B26] Huang Y. , Wang H., Lian Y., et al (2018). Upregulation of kinesin family member 4A enhanced cell proliferation via activation of Akt signaling and predicted a poor prognosis in hepatocellular carcinoma. Cell Death Dis. 9, 141. 29396392 10.1038/s41419-017-0114-4 PMC5833581

[mjab031-B27] Iorio F. , Knijnenburg T.A., Vis D.J., et al (2016). A landscape of pharmacogenomic interactions in cancer. Cell 166, 740–754. 27397505 10.1016/j.cell.2016.06.017 PMC4967469

[mjab031-B28] Ju L. , Han M., Zhao C., et al (2016). EGFR, KRAS and ROS1 variants coexist in a lung adenocarcinoma patient. Lung Cancer 95, 94–97. 27040858 10.1016/j.lungcan.2016.03.005

[mjab031-B29] Kiseljak-Vassiliades K. , Zhang Y., Kar A., et al (2018). Elucidating the role of the maternal embryonic leucine zipper kinase in adrenocortical carcinoma. Endocrinology 159, 2532–2544. 29790920 10.1210/en.2018-00310 PMC6669820

[mjab031-B30] Knijnenburg T.A. , Klau G.W., Iorio F., et al (2016). Logic models to predict continuous outputs based on binary inputs with an application to personalized cancer therapy. Sci. Rep. 6, 36812. 27876821 10.1038/srep36812 PMC5120272

[mjab031-B31] Li H. , Courtois E.T., Sengupta D., et al (2017). Reference component analysis of single-cell transcriptomes elucidates cellular heterogeneity in human colorectal tumors. Nat. Genet. 49, 708–718. 28319088 10.1038/ng.3818

[mjab031-B32] Liu J. (2018). The dualistic origin of human tumors. Semin. Cancer Biol. 53, 1–16. 30040989 10.1016/j.semcancer.2018.07.004 PMC6553492

[mjab031-B33] Malta T.M. , Sokolov A., Gentles A.J., et al (2018). Machine learning identifies stemness features associated with oncogenic dedifferentiation. Cell 173, 338–354.e15. 29625051 10.1016/j.cell.2018.03.034 PMC5902191

[mjab031-B34] Martincorena I. , Raine K.M., Gerstung M., et al (2017). Universal patterns of selection in cancer and somatic tissues. Cell 171, 1029–1041.e21. 29056346 10.1016/j.cell.2017.09.042 PMC5720395

[mjab031-B35] Mertins P. , Tang L.C., Krug K., et al (2018). Reproducible workflow for multiplexed deep-scale proteome and phosphoproteome analysis of tumor tissues by liquid chromatography-mass spectrometry. Nat. Protoc. 13, 1632–1661. 29988108 10.1038/s41596-018-0006-9 PMC6211289

[mjab031-B36] Mroz E.A. , Tward A.D., Hammon R.J., et al (2015). Intra-tumor genetic heterogeneity and mortality in head and neck cancer: analysis of data from The Cancer Genome Atlas. PLoS Med. 12, e1001786. 25668320 10.1371/journal.pmed.1001786 PMC4323109

[mjab031-B37] Nazor K.L. , Altun G., Lynch C., et al (2012). Recurrent variations in DNA methylation in human pluripotent stem cells and their differentiated derivatives. Cell Stem Cell 10, 620–634. 22560082 10.1016/j.stem.2012.02.013 PMC3348513

[mjab031-B38] Ou S.I. , Cui J., Schrock A.B., et al (2017). Emergence of novel and dominant acquired EGFR solvent-front mutations at Gly796 (G796S/R) together with C797S/R and L792F/H mutations in one EGFR (L858R/T790M) NSCLC patient who progressed on osimertinib. Lung Cancer 108, 228–231. 28625641 10.1016/j.lungcan.2017.04.003

[mjab031-B39] Ozturk K. , Dow M., Carlin D.E., et al (2018). The emerging potential for network analysis to inform precision cancer medicine. J. Mol. Biol. 430, 2875–2899. 29908887 10.1016/j.jmb.2018.06.016 PMC6097914

[mjab031-B40] Paez J.G. , Janne P.A., Lee J.C., et al (2004). EGFR mutations in lung cancer: correlation with clinical response to gefitinib therapy. Science 304, 1497–1500. 15118125 10.1126/science.1099314

[mjab031-B41] Parker J.S. , Mullins M., Cheang M.C., et al (2009). Supervised risk predictor of breast cancer based on intrinsic subtypes. J. Clin. Oncol. 27, 1160–1167. 19204204 10.1200/JCO.2008.18.1370 PMC2667820

[mjab031-B42] Pe'er D. , Hacohen N. (2011). Principles and strategies for developing network models in cancer. Cell 144, 864–873. 21414479 10.1016/j.cell.2011.03.001 PMC3082135

[mjab031-B43] Peiris-Pages M. , Martinez-Outschoorn U.E., Pestell R.G., et al (2016). Cancer stem cell metabolism. Breast Cancer Res. 18, 55. 27220421 10.1186/s13058-016-0712-6 PMC4879746

[mjab031-B44] Perou C.M. , Sorlie T., Eisen M.B., et al (2000). Molecular portraits of human breast tumours. Nature 406, 747–752. 10963602 10.1038/35021093

[mjab031-B45] Platzer A. , Perco P., Lukas A., et al (2007). Characterization of protein-interaction networks in tumors. BMC Bioinformatics 8, 224. 17597514 10.1186/1471-2105-8-224 PMC1929125

[mjab031-B46] Pleasance E.D. , Cheetham R.K., Stephens P.J., et al (2010). A comprehensive catalogue of somatic mutations from a human cancer genome. Nature 463, 191–196. 20016485 10.1038/nature08658 PMC3145108

[mjab031-B47] Prahallad A. , Sun C., Huang S., et al (2012). Unresponsiveness of colon cancer to BRAF(V600E) inhibition through feedback activation of EGFR. Nature 483, 100–103. 22281684 10.1038/nature10868

[mjab031-B48] Prasetyanti P.R. , Medema J.P. (2017). Intra-tumor heterogeneity from a cancer stem cell perspective. Mol. Cancer 16, 41. 28209166 10.1186/s12943-017-0600-4 PMC5314464

[mjab031-B49] Reiter J.G. , Baretti M., Gerold J.M., et al (2019). An analysis of genetic heterogeneity in untreated cancers. Nat. Rev. Cancer 19, 639–650. 31455892 10.1038/s41568-019-0185-x PMC6816333

[mjab031-B50] Salvucci M. , Zakaria Z., Carberry S., et al (2019). System-based approaches as prognostic tools for glioblastoma. BMC Cancer 19, 1092. 31718568 10.1186/s12885-019-6280-2 PMC6852738

[mjab031-B51] Shibue T. , Weinberg R.A. (2017). EMT, CSCs, and drug resistance: the mechanistic link and clinical implications. Nat. Rev. Clin. Oncol. 14, 611–629. 28397828 10.1038/nrclinonc.2017.44 PMC5720366

[mjab031-B52] Sorlie T. , Perou C.M., Tibshirani R., et al (2001). Gene expression patterns of breast carcinomas distinguish tumor subclasses with clinical implications. Proc. Natl Acad. Sci. USA 98, 10869–10874. 11553815 10.1073/pnas.191367098 PMC58566

[mjab031-B53] Teschendorff A.E. , Enver T. (2017). Single-cell entropy for accurate estimation of differentiation potency from a cell's transcriptome. Nat. Commun. 8, 15599. 28569836 10.1038/ncomms15599 PMC5461595

[mjab031-B54] Teschendorff A.E. , Severini S. (2010). Increased entropy of signal transduction in the cancer metastasis phenotype. BMC Syst. Biol. 4, 104. 20673354 10.1186/1752-0509-4-104 PMC2925356

[mjab031-B55] Teschendorff A.E. , Sollich P., Kuehn R. (2014). Signalling entropy: a novel network-theoretical framework for systems analysis and interpretation of functional omic data. Methods 67, 282–293. 24675401 10.1016/j.ymeth.2014.03.013

[mjab031-B56] Teufel M. , Seidel H., Kochert K., et al (2019). Biomarkers associated with response to regorafenib in patients with hepatocellular carcinoma. Gastroenterology 156, 1731–1741. 30738047 10.1053/j.gastro.2019.01.261

[mjab031-B57] Visvader J.E. , Lindeman G.J. (2012). Cancer stem cells: current status and evolving complexities. Cell Stem Cell 10, 717–728. 22704512 10.1016/j.stem.2012.05.007

[mjab031-B58] Vogelstein B. , Papadopoulos N., Velculescu V.E., et al (2013). Cancer genome landscapes. Science 339, 1546–1558. 23539594 10.1126/science.1235122 PMC3749880

[mjab031-B59] Wang J. , Wang Y., Shen F., et al (2018). Maternal embryonic leucine zipper kinase: a novel biomarker and a potential therapeutic target of cervical cancer. Cancer Med. 7, 5665–5678. 30334367 10.1002/cam4.1816 PMC6246930

[mjab031-B60] Wells A. (1999). EGF receptor. Int. J. Biochem. Cell Biol. 31, 637–643. 10404636 10.1016/s1357-2725(99)00015-1

[mjab031-B61] West J. , Bianconi G., Severini S., et al (2012). Differential network entropy reveals cancer system hallmarks. Sci. Rep. 2, 802. 23150773 10.1038/srep00802 PMC3496163

[mjab031-B62] Woodard G.A. , Jones K.D., Jablons D.M. (2016). Lung cancer staging and prognosis. Cancer Treat. Res. 170, 47–75. 27535389 10.1007/978-3-319-40389-2_3

[mjab031-B63] Woodburn J.R. (1999). The epidermal growth factor receptor and its inhibition in cancer therapy. Pharmacol. Ther. 82, 241–250. 10454201 10.1016/s0163-7258(98)00045-x

[mjab031-B64] Yau C. , Esserman L., Moore D.H., et al (2010). A multigene predictor of metastatic outcome in early stage hormone receptor-negative and triple-negative breast cancer. Breast Cancer Res. 12, R85. 20946665 10.1186/bcr2753 PMC3096978

[mjab031-B65] Yu Y.P. , Landsittel D., Jing L., et al (2004). Gene expression alterations in prostate cancer predicting tumor aggression and preceding development of malignancy. J. Clin. Oncol. 22, 2790–2799. 15254046 10.1200/JCO.2004.05.158

[mjab031-B66] Zheng H. , Pomyen Y., Hernandez M.O., et al (2018). Single-cell analysis reveals cancer stem cell heterogeneity in hepatocellular carcinoma. Hepatology 68, 127–140. 29315726 10.1002/hep.29778 PMC6033650

