# Supplementary material for: Pan-cancer network disorders revealed by overall and local signaling entropy
Source: J Mol Cell Biol. 2021 Jun 7;13(9):622–35. doi: 10.1093/jmcb/mjab031 (PMC8648393; doi:10.1093/jmcb/mjab031)
Supplement: mjab031_Supplementary_Data [file mjab031_supplementary_data.pdf]

Supplementary Figures

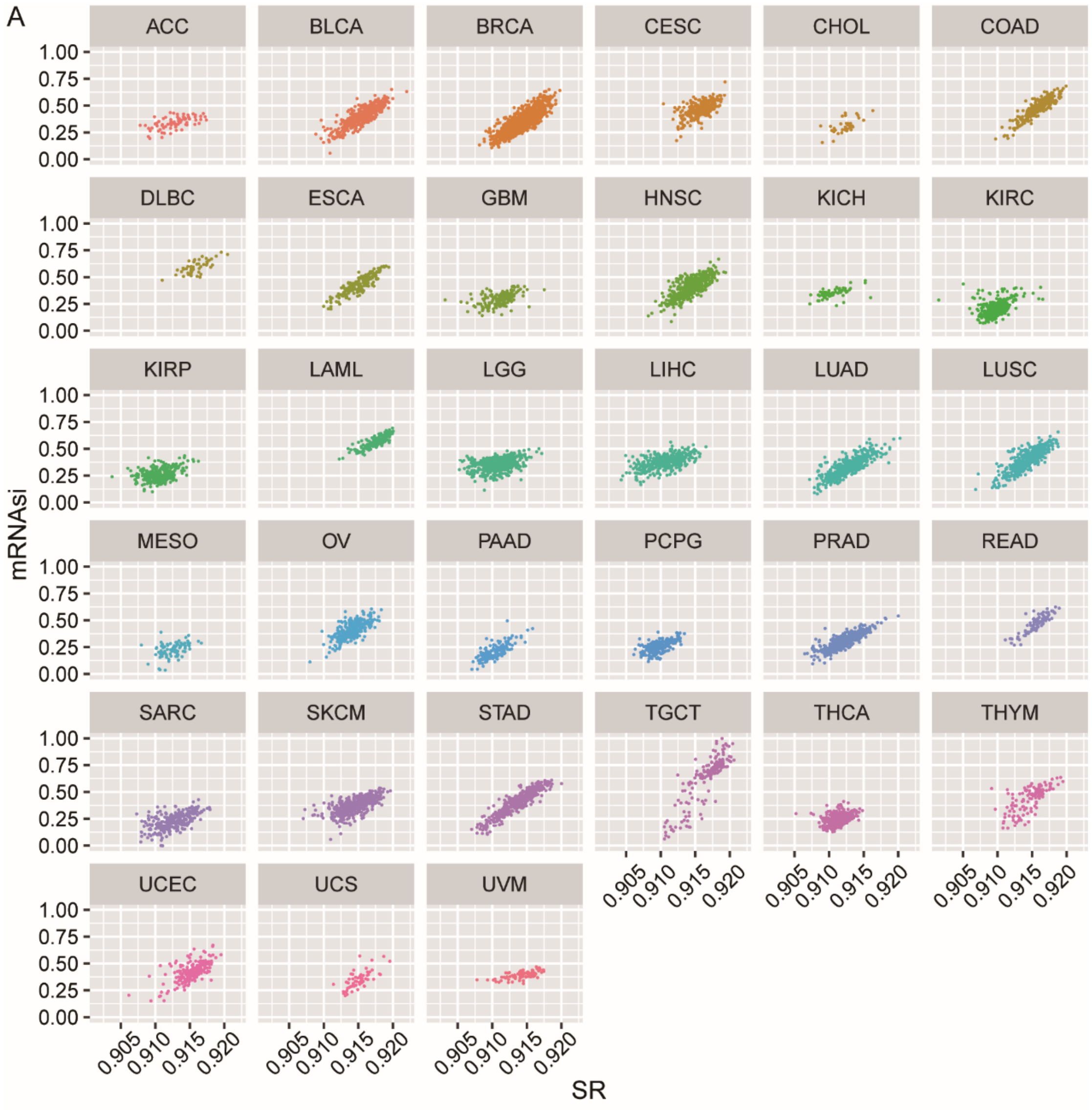

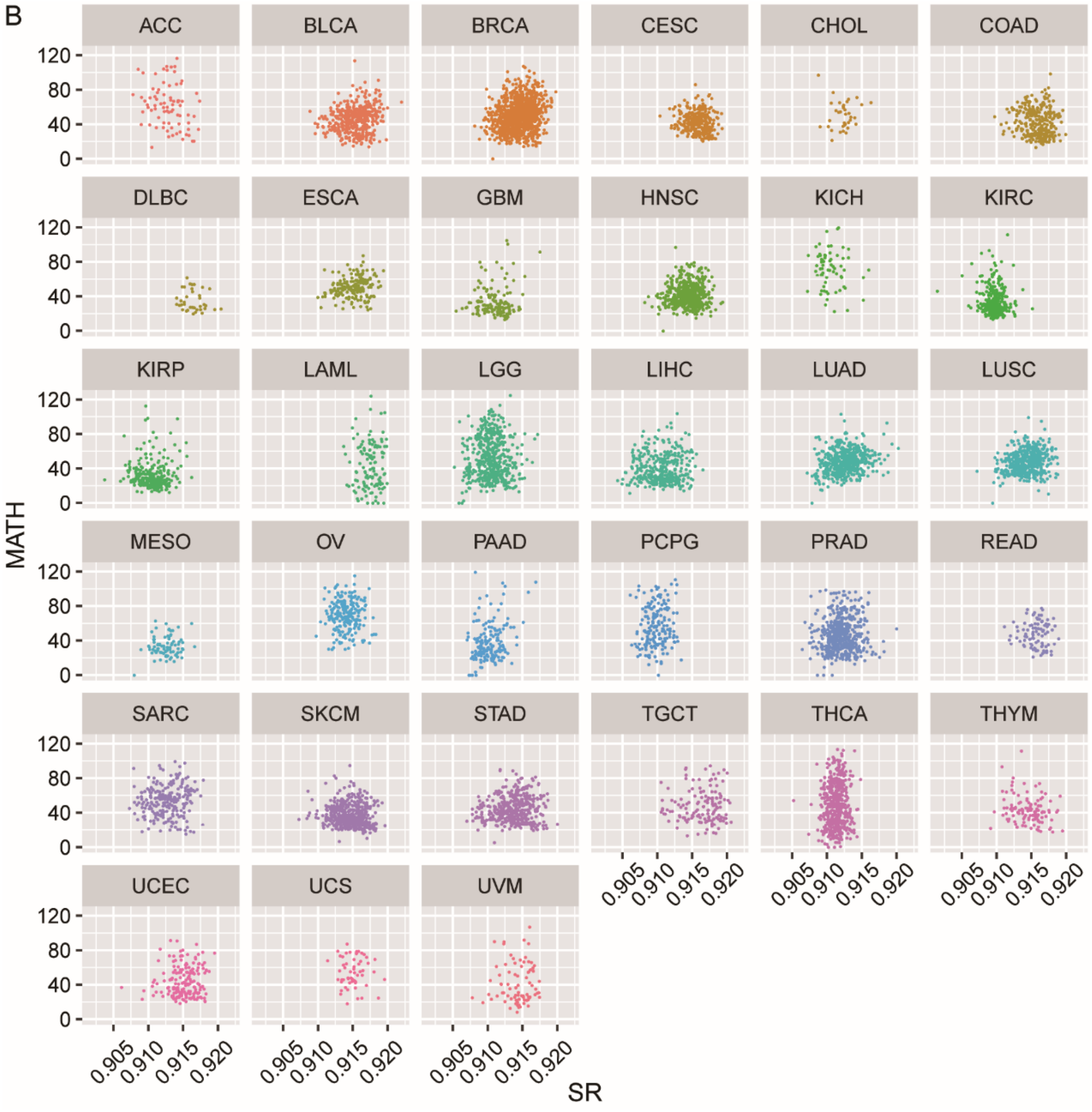

Supplementary Figure S1

(**A**) The correlation indices between signaling entropy and mRNAsi in 33 tumor types of TCGA database.

(**B**) The correlation indices between signaling entropy and MATH in 33 tumor types of TCGA database.

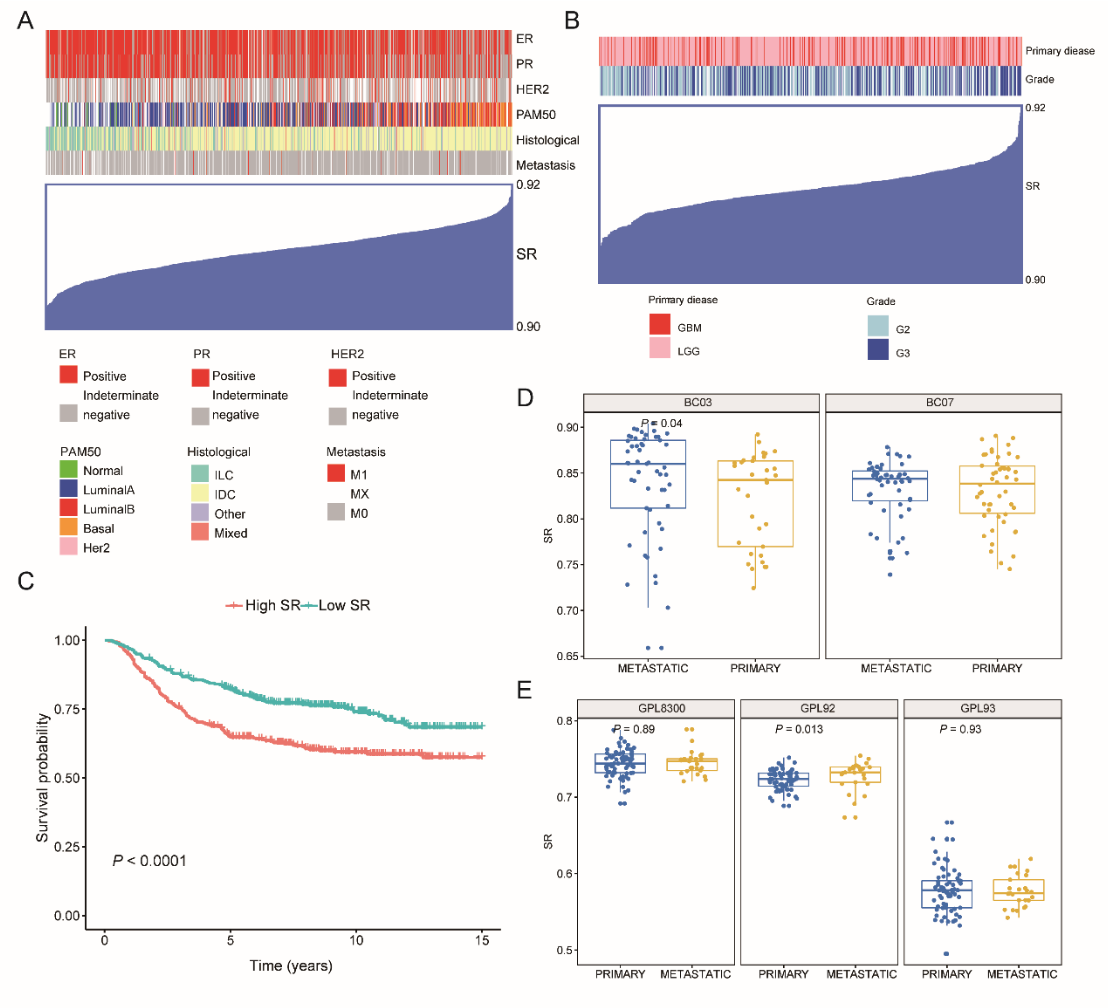

Supplementary Figure S2

(**A**) An overview of the association between clinical characteristics and signaling entropy in BRCA. Columns represent samples sorted by signaling entropy from low to high.

(**B**) An overview of the association between clinical characteristics and Signaling Entropy in glioma, GBM and LGG. Columns represent samples sorted by signaling entropy from low to high.

(**C**) Survival of breast cancers of high signaling entropy (red) compared with breast cancers of low signaling entropy (green).

(**D**) Signaling entropy is higher in metastases cells in comparison to the cells in primary sites of breast cancer.

(**E**) Signaling entropy is higher in cancer metastases in comparison to the primary tumors of prostate cancer.

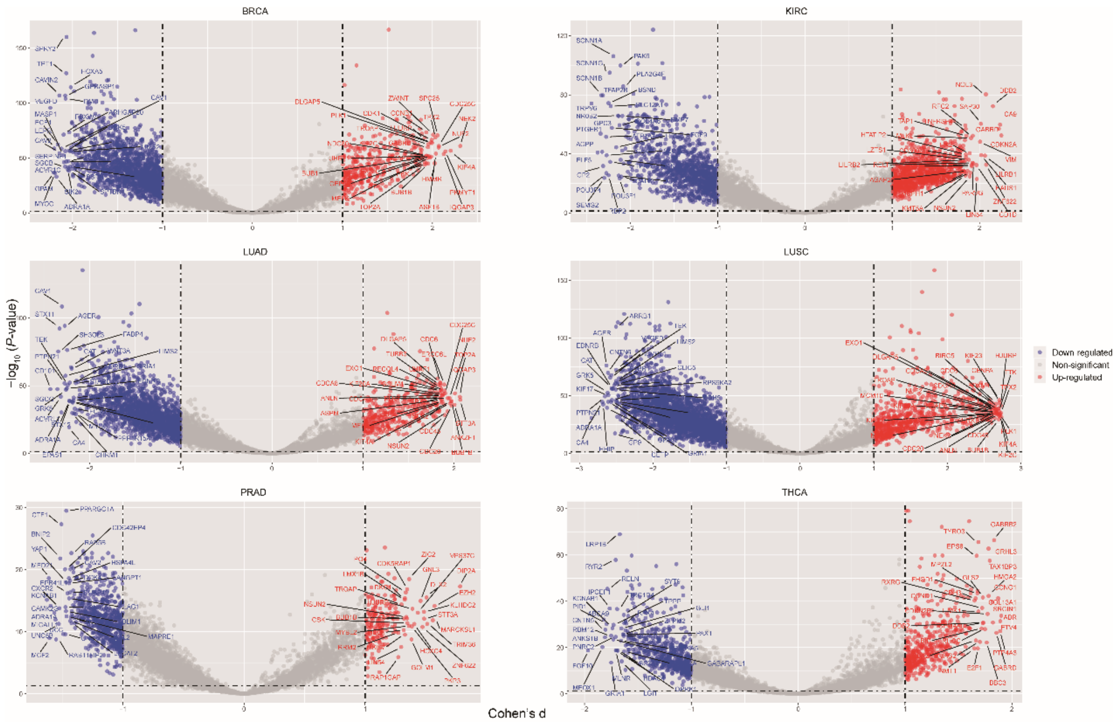
BRCA
KIRC
LUAD
LUSC
PRAD
THCA
$-\log_{10}$ (P-value)
Cohen's d
Down regulated
Non-significant
Up-regulated

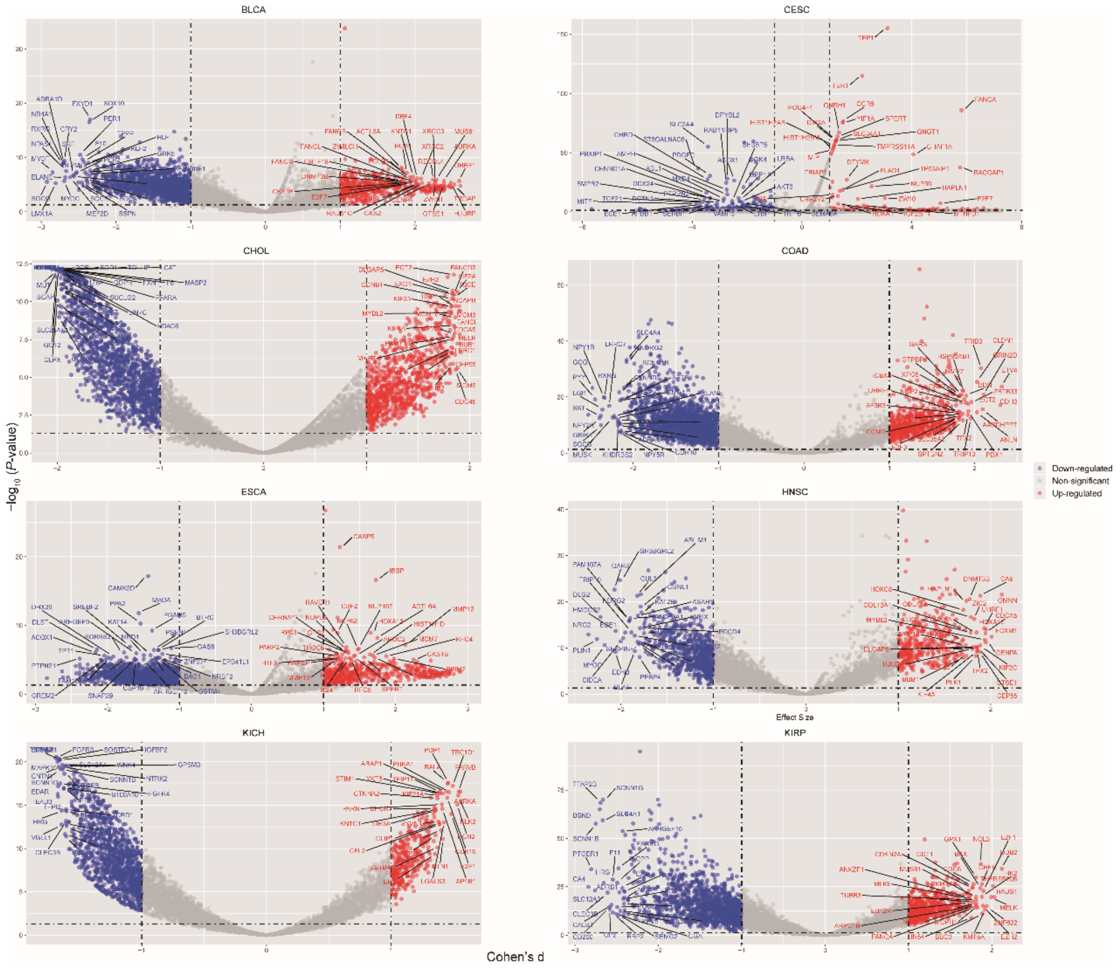
BLCA
CESC
CHOL
COAD
ESCA
HNSC
KICH
KIRP
$-\log_{10}$ (P-value)
Cohen's d
Effect Size
Down-regulated
Non-significant
Up-regulated

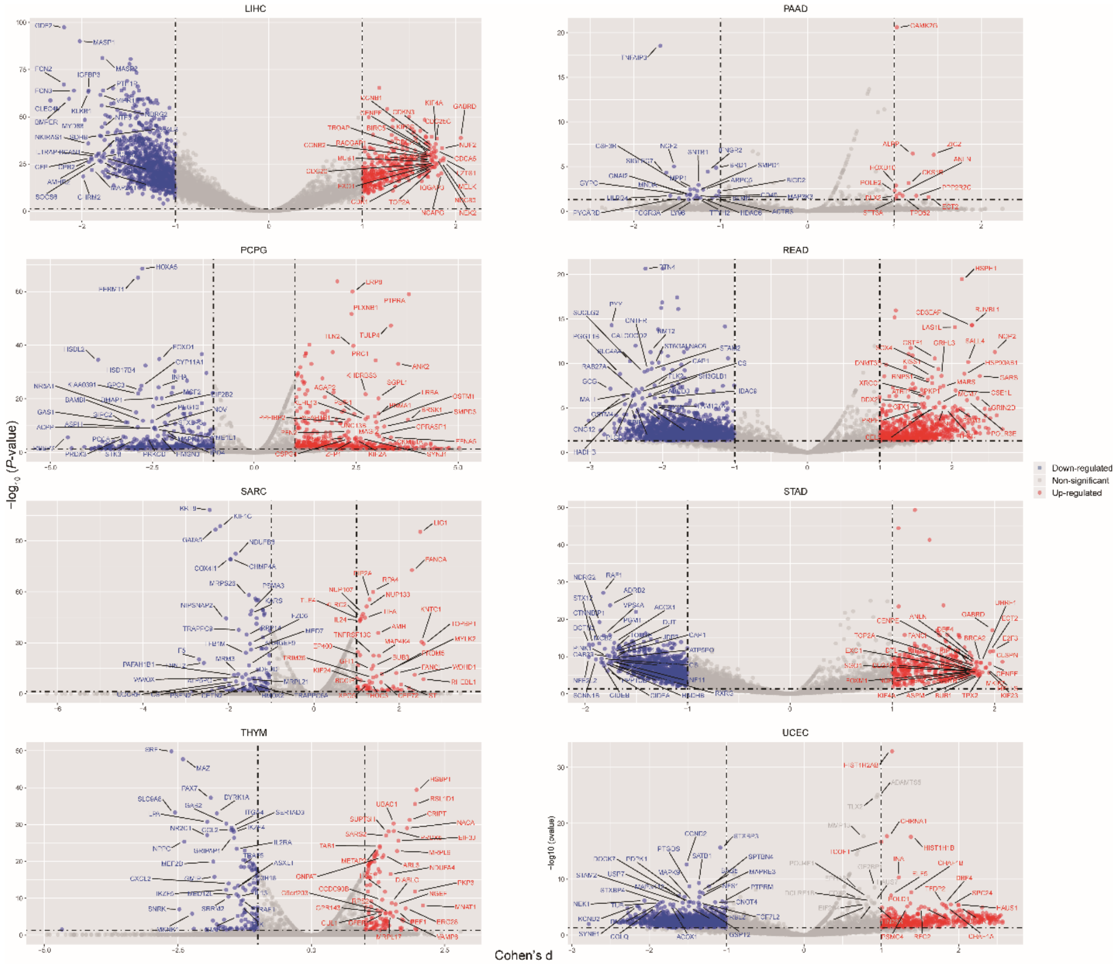

Supplementary Figure S3

Volcano plots of differentially expressed LSR between tumor and normal in all 22 cancer types displays effect size against -log10(P-value) (P-value adjusted by fdr method from the t-test).

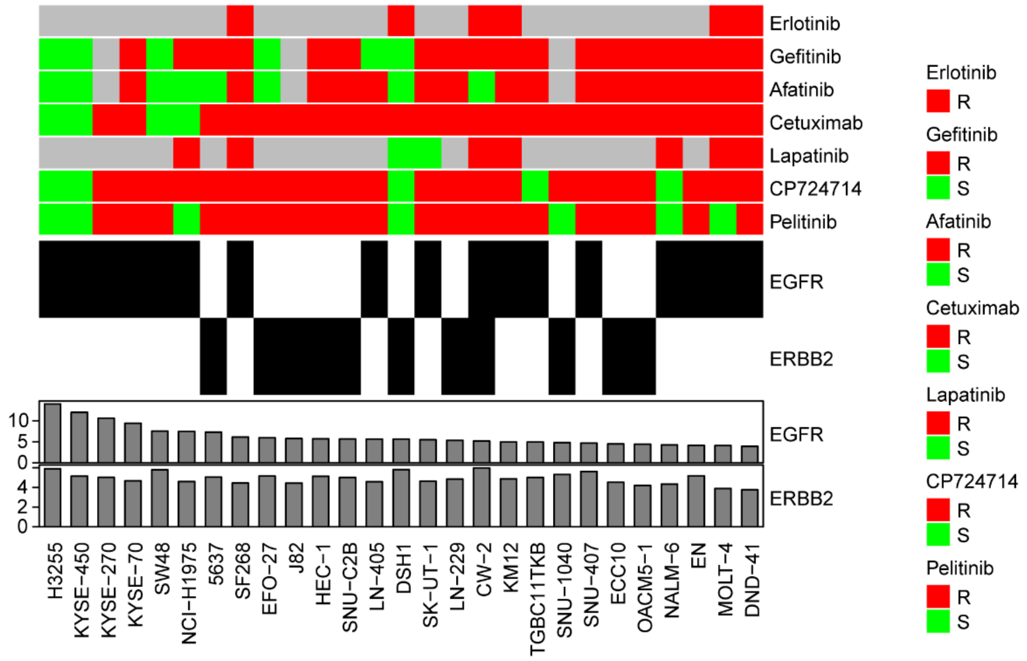

Supplementary Figure S4

Drug response associated with mutation and local signaling entropy ratio. The first seven rows represent drug response, red means resistance, green means sensitive. Then 2 rows show the mutation situation, black means there is a mutation. The last 2 rows are bar plots, which show the local signaling entropy ratio*10000.

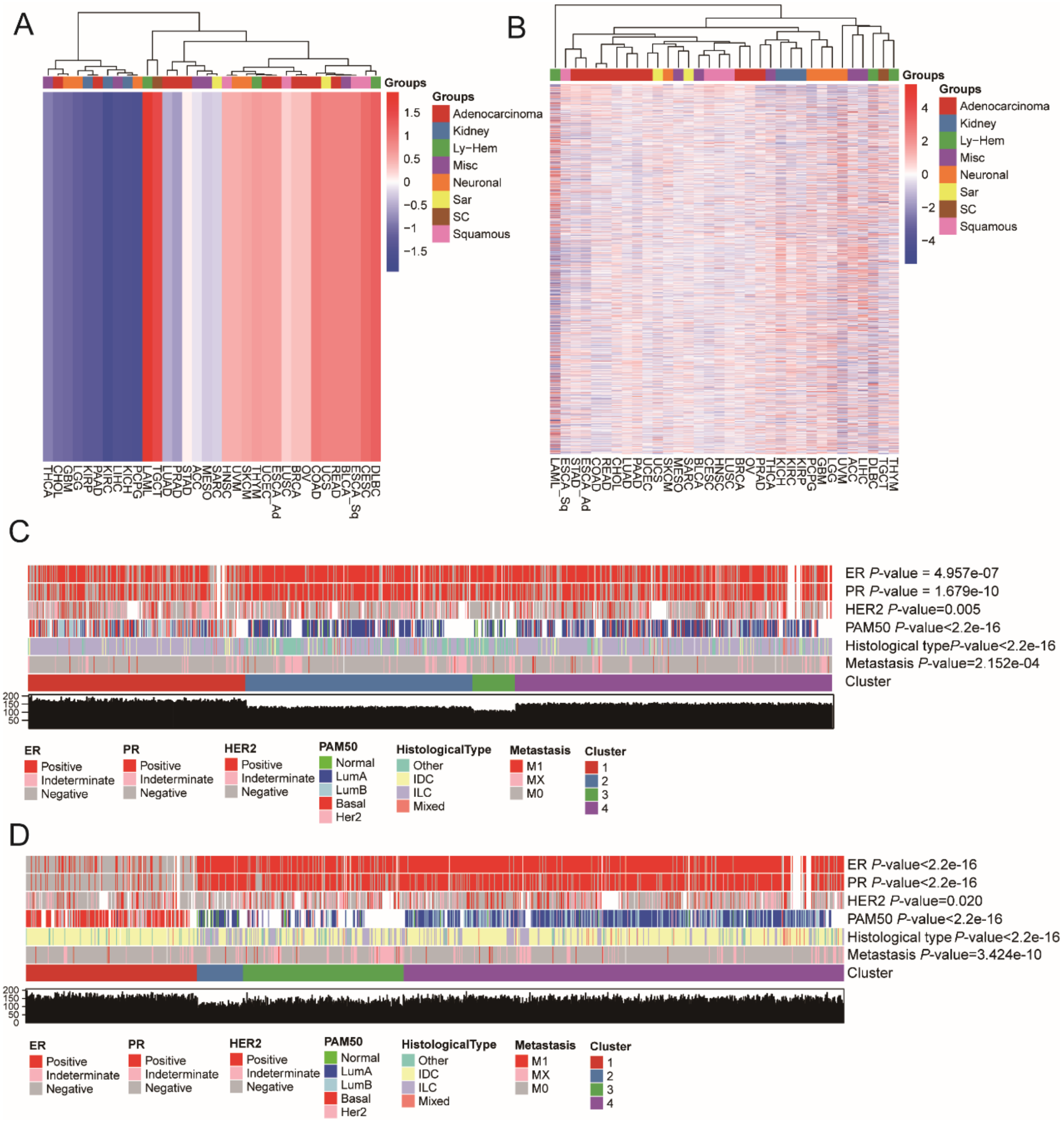

Supplementary Figure S5

(**A**) Hierarchical clustering using signaling entropy of 34 cancer types.

(**B**) Hierarchical clustering using local signaling entropy matrix of 34 cancer types.

(**C**) 4 clusters obtained by k-means clustering using signaling entropy of BRCA samples were significantly related to various clinical characteristics.

(**D**) 4 clusters obtained by k-means clustering using local signaling entropy matrix of BRCA samples were significantly related to various clinical characteristics.

Bar plot shows (signaling entropy-0.9) *10000.
